# Investigating the seasonality pattern of ultrasound requests and its association with photoperiod: A cross-sectional retrospective study

**DOI:** 10.1097/MD.0000000000044695

**Published:** 2025-09-26

**Authors:** Wael A. Ageeli, Ali Y. Swedi, Turkey A. Refaee, Ali M. Tawhari, Shaima A. Shanaq, Asmahan Y. Alhazmi, Abeer H. Maashi, Elaf A. Alsalem, Marwa A. Atiah, Ali A. Hommadi, Mohammed O. Kuliby, Ali S. Alyami, Bandar A. Alwadani, Nouf Abuhadi, Yahia Madkhali, Naif A. Majrashi

**Affiliations:** a Diagnostic Radiography Technology (DRT) Department, Faculty of Nursing and Health Sciences, Jazan University, Jazan, Saudi Arabia; b Jazan Health Cluster Samtah, General Hospital, Jazan, Saudi Arabia.

**Keywords:** cosinor, order, photoperiod, priority, seasonality, ultrasound, US requests

## Abstract

Seasonal variations in healthcare utilization, particularly in medical imaging services such as ultrasound (US), are gaining interest because of their potential links with environmental factors such as photoperiod. Although previous research has documented seasonal changes in healthcare activities, the seasonality of imaging requests remains underexplored, which could impact resource planning. This study aimed to investigate the seasonal patterns of US requests and their association with the photoperiod. We analyzed a 3-year dataset of 20,142 US requests from Samtah General Hospital in Jazan. The photoperiod, defined as daily daylight hours, was calculated using the “season” package in R based on the coordinates of the hospital. Statistical analyses included Cox generalized linear regression to assess seasonality, multinomial regression to explore the relationship between US requests and photoperiod, and binomial logistic regression to evaluate the association between order priority and photoperiod. Significant seasonality was noted for all US requests, peaking in August and November. The subtypes varied in seasonal amplitudes, with abdominal and pelvic scans exhibiting the highest fluctuations. A positive correlation was identified between the photoperiod and the 3D US scan subtype. Additionally, the US order priority showed seasonal patterns, with routine cases peaking in December and urgent cases in September. This study highlights the seasonality of ultrasound requests and their correlation with the photoperiod in the Jazan region. Understanding these patterns is essential for effective health care planning and resource allocation. Future research should integrate more contextual factors to clarify the relationship between seasonality and diagnostic practices, optimize healthcare services, and improve patient outcomes in Jazan.

## 1. Introduction

The seasonality of health conditions has gained interest in medical research because of its impact on disease epidemiology, which affects the occurrence, severity, and manifestation of various conditions.^[1–4]^ These fluctuations are influenced by a multitude of factors including environmental cues, genetic predispositions, and behavioral patterns. One aspect that has garnered attention is the potential influence of photoperiod, which refers to the length of daylight exposure during a given day on certain medical practices and procedures. Alterations in photoperiod are known to affect biological rhythms, such as sleep-wake cycles, hormone regulation, mood, and brain morphology.^[5,6]^ These changes may affect healthcare-seeking behaviors and medical service demand by interacting with cultural and societal factors.

Previous research has revealed seasonal changes in healthcare use, such as trends in emergency department visits, hospital admissions, and outpatient clinic consultations.^[7]^ Such variations are frequently related to changes in weather patterns, infectious illness incidence, and cultural behaviors. In the field of medical imaging, understanding the seasonal patterns of imaging requests, such as ultrasound (US) examinations, remains relatively unknown^[1,2]^ and could have important implications for resource planning and optimization.

US imaging is a key diagnostic tool because of its noninvasive nature and broad applicability.^[8]^ The demand for US examinations can be influenced by factors, such as the prevalence of specific health conditions, referral patterns, and patient preferences. However, the potential relationship between the seasonality of US requests and their association with photoperiod has not been extensively explored in the literature.

Given the potential influence of seasonal changes in emergency department visits, hospital admissions, outpatient clinic consultations, and photoperiod on human physiology and behavior, it is plausible that the seasonality of diagnostic imaging, including US requests, may be influenced by changes in daylight exposure over the course of the year. Understanding this relationship can offer insights into healthcare utilization and aid in resource planning. The aim of this cross-sectional study was to investigate the seasonality pattern of US requests and its association with photoperiod in Jazan, which offers a unique setting to study the seasonality of abnormal US findings because its tropical climate, diverse geography, and ethnically heterogeneous population provide an intriguing context to explore how seasonal variations may influence disease patterns and prevalence.^[9]^ By analyzing the trends in US requests and correlating them with local photoperiod data, this study seeks to contribute to the growing body of knowledge on seasonal variations in medical imaging practices and their potential drivers.

## 2. Methods

### 2.1. Imaging (US) characteristics

US requests were categorized based on the type of scan conducted for specific organs. Table 1 provides an overview of the study characteristics related to US requests. The distribution of US scans varied across different types, including obstetric, cranial, Doppler lower limb, Doppler (general), Doppler upper limb, neck, breast, and others. US requests were further categorized based on their status, distinguishing between routine and urgent requests. All Participants underwent US at a specific location (Samtah, Jazan). All US requests for this study were taken from 2020 to 2022. All participants lived in Samtah, Saudi Arabia. The latitude and longitude information of the scanning location, Samtah, were used to calculate the photoperiod, which represents the hours of daylight on the day of the scan. The “season” package in R (https://www.r-project.org/) was utilized to derive photoperiod by subtracting the sunset time from the sunrise time on the day of the scan. Ethical approval was obtained from the Jazan Health Ethics Committee (no. 2413).

**Table 1:** Characteristics of US requests and participants.

Variables	(Number, %)	
Total US requests	20,142 (100%)	
Female US requests	13,687 (67.9%)	
Male US requests	6455 (32.1%)	
Types of US scans		
US_Obstetrics	4030 (20.02%)	
US_Cranial	72 (0.36%)	
US_Doppler_lower_limbs	443 (2.20%)	
US_Doppler	111 (0.55%)	
US_Doppler_upper_limbs	77 (0.38%)	
US_Neck	333 (1.65%)	
US_Breast	576 (2.86%)	
Ultrasound_3D	233 (1.16%)	
US_Pregnancy_fetal_anomaly	18 (0.09%)	
US_Anterior_Abdominal_Wall	31 (0.15%)	
US_Prostate	798 (3.97%)	
US_Soft_tissue	41 (0.20%)	
US_Doppler_carotid	39 (0.19%)	
US_Doppler_testes	73 (0.36%)	
US_Legs	15 (0.07%)	
US_Urinary_tract	13 (0.06%)	
US_Hips	17 (0.08%)	
US_Pelvis_transvaginal	20 (0.10%)	
US_Gallbladder	12 (0.06%)	
US_Doppler_renal	12 (0.06%)	
US_Kidney	14 (0.07%)	
US_Pelvis	433 (2.15%)	
US_Adrenals	15 (0.07%)	
US_Axilla	23 (0.11%)	
US_Abdomen_and_pelvis	4784 (23.77%)	
US_Abdomen_and_renal_tract	2114 (10.51%)	
US_Testes	453 (2.25%)	
US_Abdomen	5130 (25.49%)	
US_Thyroid	140 (0.70%)	
US_Groin_and_inguinal_region	72 (0.36%)	
US order priority		
Routine requests	11,406 (56.6%)	
Urgent requests	8736 (43.4%)	

	Mean	SD
Age (yr)	38.4	17.5
Photoperiod in hours	12.1	0.678
Cosine MOS transformation	0.046	0.005
Sin MOS transformation	−0.080	0.004

MOS = month of scan, N = number, SD = standard deviation, Sin = Sine function, US = ultrasound.

### 2.2. Photoperiod calculation

Photoperiod in hours of daylight on the day of scan was derived from the latitude and longitude information of the scanning location (Samtah, Jazan) determined by the “season” package in R (https://www.r-project.org/). The photoperiod in hours was calculated by subtracting sunset from sunrise on the day of the scan. The photoperiod served as a continuous measure of day length.

### 2.3. Statistical analyses

Statistical analyses were performed using R software, with a significance level (alpha) set at *P* = .05 for all tests. To address the potential for type I (false positive) statistical errors, the results were adjusted for multiple comparisons using the standard Bonferroni correction criterion.^[10]^ To investigate the seasonality of US requests, a cosinor-generalized linear regression (GLM) analysis was conducted. The analysis examined 2 key variables: the number of US requests per month, which represented the total number of US examinations performed by organ type, and the number of routine and urgent US requests per month, analyzed separately. This analysis incorporated sine and cosine functions, with month serving as the time variable.^[11,12]^ Additionally, cosine and sine transformations of the month of scan were computed to evaluate the seasonal pattern in the number and priority of US requests using the following formulas:


$$\begin{array}{l} Sin=\left(2*\;\pi \;\left(M-1\right)/12\right) \end{array}$$

$$\begin{array}{l} Cos=\left(2*\;\pi \;\left(M-1\right)/12\right) \end{array}$$

where M is the month of scan (integer numbers from 1 to 12). To evaluate the sinusoidal nature of the seasonal pattern in US scans (the number of US scans), we compared a model that included sine and cosine transformations of the month, as well as age and sex as covariates, with models that excluded these monthly transformations.^[13]^ The significance of seasonality or an improved model fit was determined based on 2 specific criteria. First, the sine and/or cosine (cosinor) terms needed to be statistically significant (*P* < .05), with an amplitude significantly greater than zero. Second, the model including the cosinor terms should have a lower Akaike information criterion, which is a useful statistic for model selection.^[13,14]^ The amplitude of the cosinor model (or curve) was calculated as


$$\begin{array}{l} \sqrt{\beta^{2}\;\;\;+\;\;\;\gamma^{2}} \end{array}$$

where β and γ are the cosine and sine generalized regression coefficients, respectively. Finally, the acrophase (φ; peak of the cosinor model) in the month of the scan was calculated as follows:


$$\begin{array}{l} \;\varphi \;=12*\frac{tan\bar{1\left(\frac{\gamma }{\beta }\right)}}{2\pi }+1\;\;\; \end{array}$$

**Figure 1.**
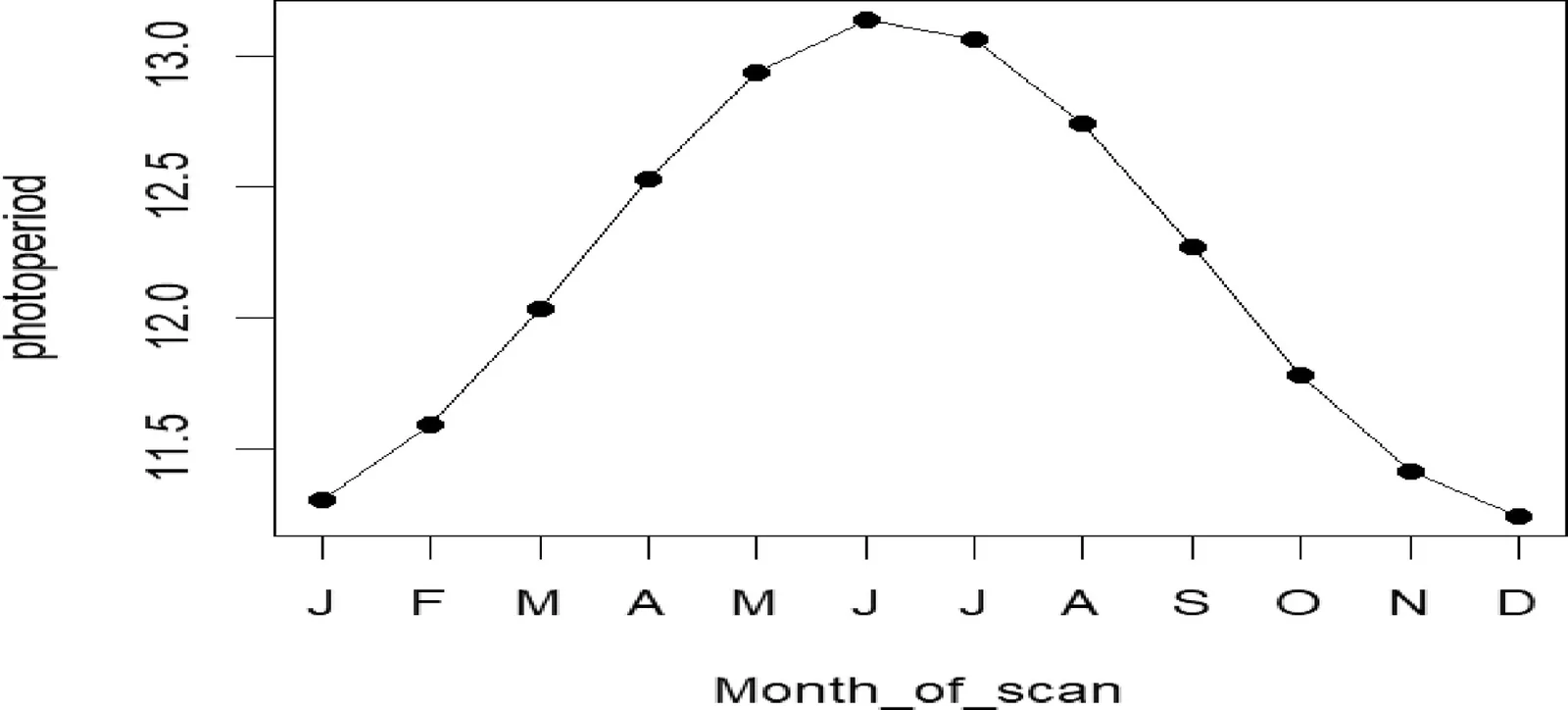
The seasonal pattern of photoperiod, the hours of daylength. The data has been fitted with a cosinor model to demonstrate the sinusoidal pattern and illustrate the changes in daylength throughout the seasons.

To assess the seasonal pattern of photoperiod, which measures day length, a sinusoidal pattern analysis was conducted. No transformations were required, as the photoperiod naturally exhibited a sinusoidal pattern (see Fig. 1). Consequently, photoperiod was used as the time variable in all regression analyses, replacing transformed months as it inherently captured the seasonal pattern. A multinomial regression model was used to investigate the relationship between US requests by scan type (representing specific organs, such as the abdomen, prostate, and breasts) and photoperiod. Additionally, a binominal regression model was used to assess the relationship between US order priority (routine or urgent) and photoperiod. Likelihood ratio tests showed that US scans were over-dispersed, with the variance exceeding the mean. This test evaluates the model fit in binominal and multinomial logistic regressions, measuring the improvement when independent variables are included. Covariates such as age and sex were added to the model.

## 3. Results

Characteristics of US Requests and Participants

This cross-sectional study at the Samtah General Hospital (SGH) analyzed 20,142 US imaging requests, with 67.9% of females and 32.1% of males. Table 1 lists the US scan types and their frequencies, with “US_Abdomen” being the most common (25.49%), followed by “US_Abdomen_and_pelvis” (23.77%) and “US_Obstetrics” (20.02%). The participants’ ages ranged from 2 to 104 years, with a mean age of 38.4 years (SD = 17.5). All scans were performed between January 2020 and December 2022 and the dates were recorded. The participants were evenly distributed between the northern and southern regions of the scanning center, averaging 25 km from the center. The photoperiod for each scan date was measured based on the location of the scanning center chosen for minimal distance variation and limited photoperiod variability. Figure 1 illustrates a sinusoidal photoperiod pattern, ranging from 11.1 hours in winter to 13.5 hours in summer. The number of US scans varied throughout the year from 12 to 5130. Detailed demographic characteristics related to the scan types are presented in Table 1.

### 3.1. Tests of seasonality of US scan requests

**Figure 2.**
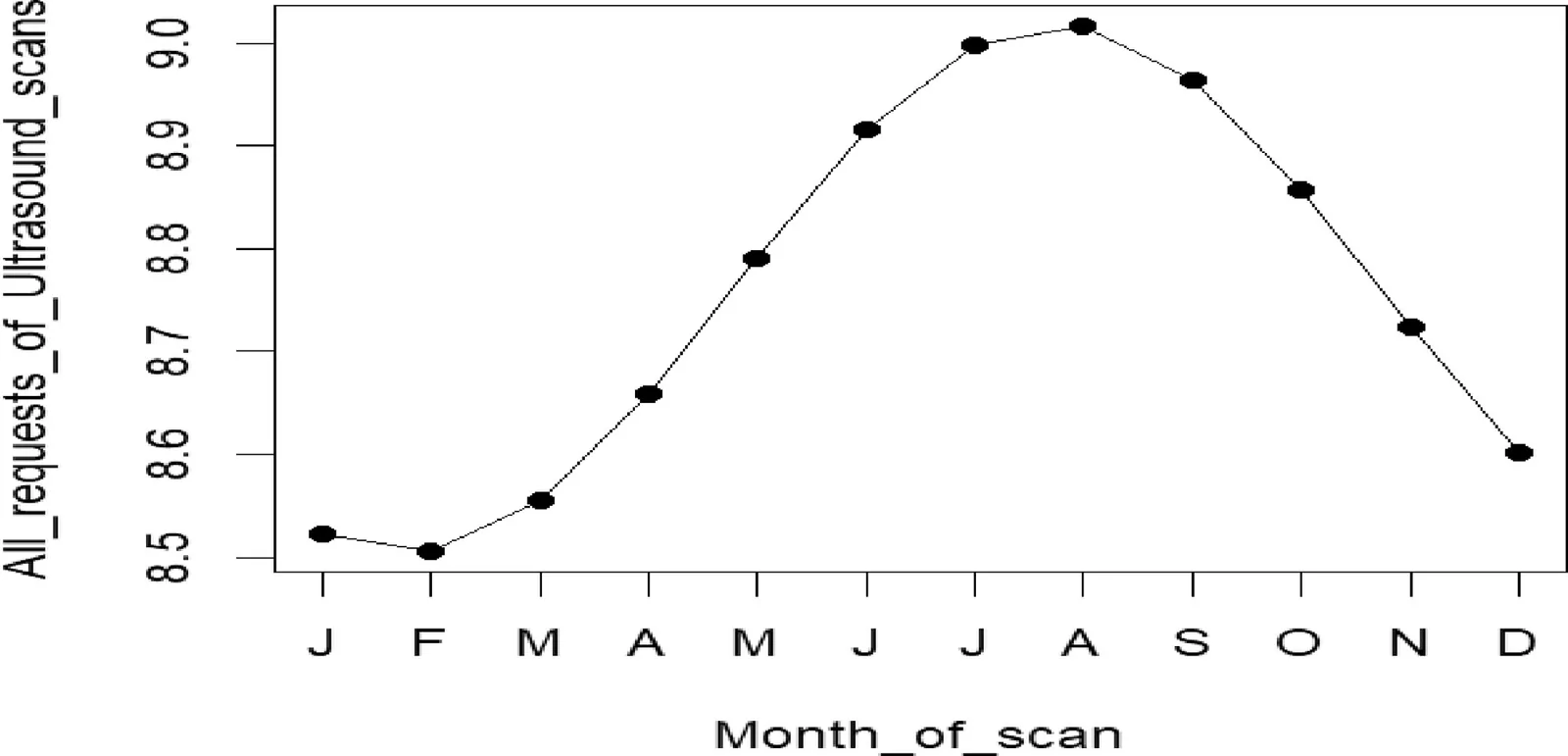
The seasonal pattern of all US scan requests over the past 3 years. The data has been fitted with a cosinor model to depict the sinusoidal pattern and highlight the variations in scan requests throughout the different seasons.

**Figure 3.**
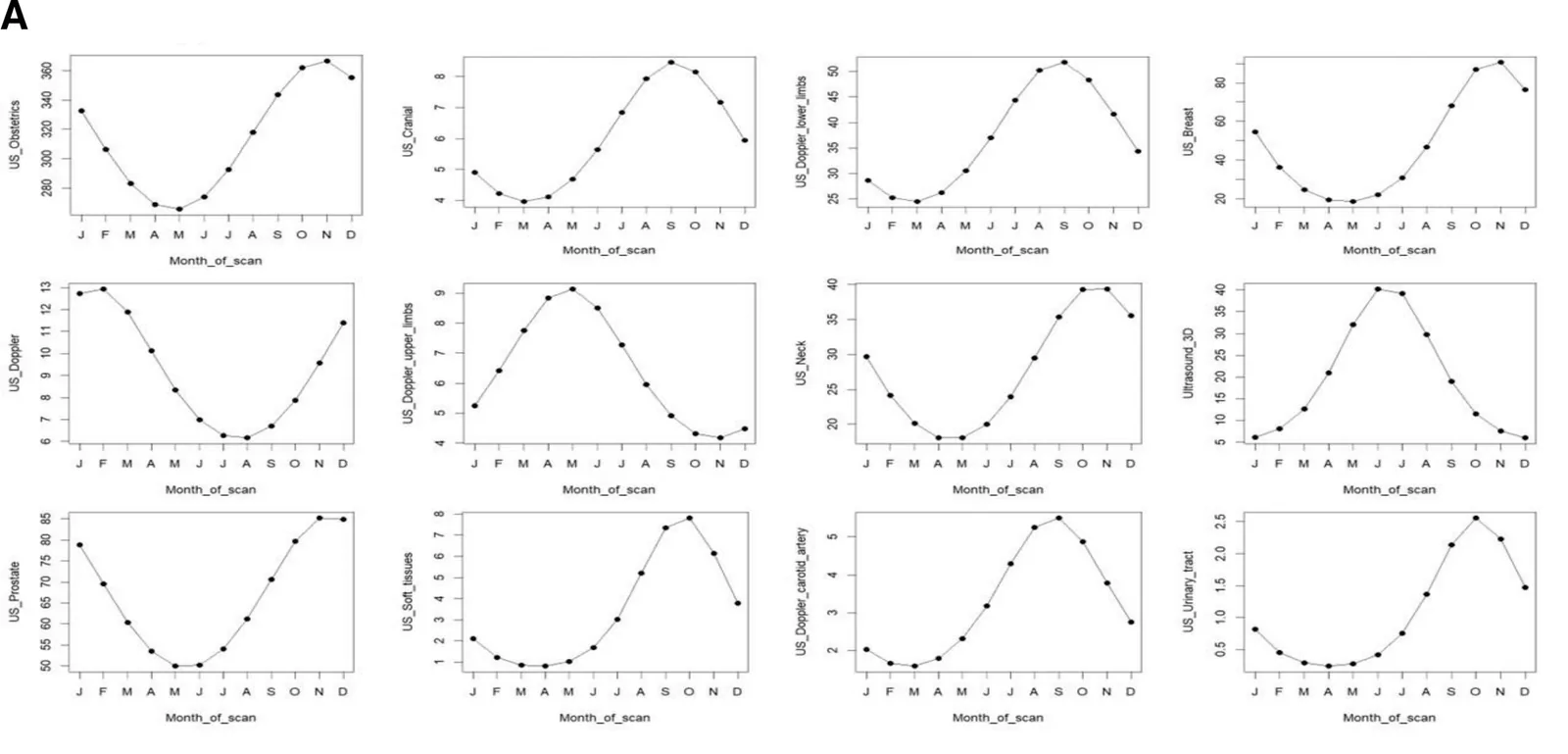
The seasonal pattern of subtypes of US requests over the past 3 years. The data has been fitted with a cosinor model to visualize the sinusoidal pattern in the different subtypes throughout the seasons.

**Figure 4.**
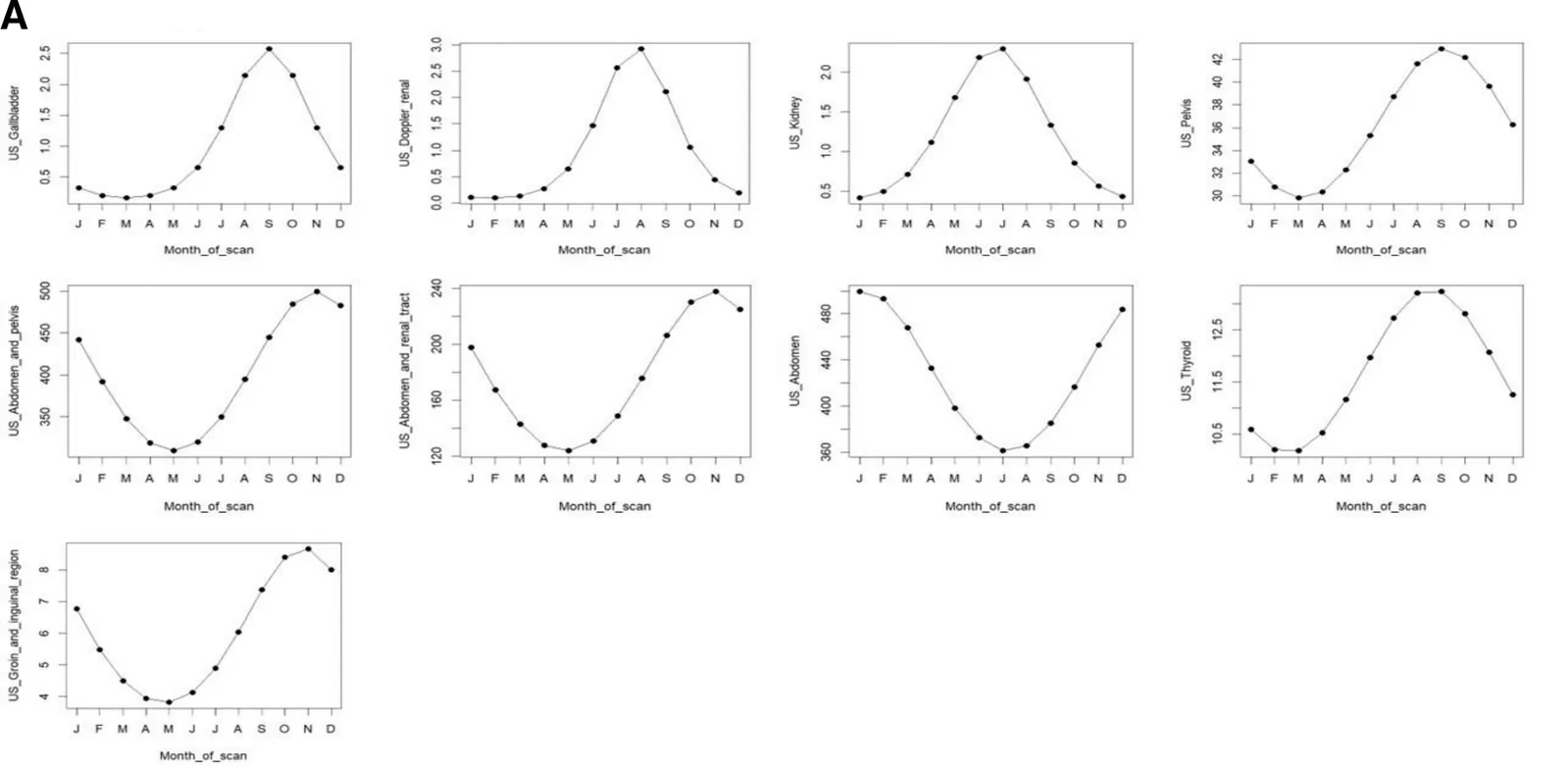
The seasonal pattern of subtypes of US requests over the past 3 years. The data has been fitted with a cosinor model to visualize the sinusoidal pattern in the different subtypes throughout the seasons.

A cosinor GLM analysis was conducted to examine sinusoidal patterns in 2 contexts: all US requests over the past 3 years and individual subtypes of US requests. The goal was to determine whether including sine and/or cosine transformations of the month of scan improved the model fit compared to models without these transformations. Significant cosine terms were found for all US requests, with a peak (acrophase) in August (Fig. 2). The highest amplitude was observed in the US abdominal and pelvic requests, followed by the US abdominal and renal tract, routine abdominal, and prostate requests (Table 2). For each subtype of US scan request, we analyzed the seasonality and the impact of sine and/or cosine transformations. Significant cosinor terms were identified for several subtypes, including US obstetrics, cranial, and general Doppler (Figs. 3 and 4). Acrophase peaks varied from January to November. The highest amplitude remained in the US abdominal and pelvic requests, followed by the abdominal and renal tract, routine abdominal, and prostate requests (Table 2). While most subtypes showed seasonality, exceptions included US pregnancy fetal anomaly requests peaking in August, anterior abdominal wall requests in February, and several others peaking in various months.

**Table 2:** Cosinor parameters and model for subtypes of US requests.

Outcome	Multivariate generalized regression estimates						Acrophase	Amplitude
US requests	Cosine coefficient	(SE)	*P*	Sine coefficient	(SE)	*P*	(Month)	
US_Obstetrics	0.064	0.023	.005	−0.149	0.035	<.001	November	71.7
US_Cranial	−0.166	0.068	.323	−0.341	0.170	.045	September	2.67
US_Doppler_lower_limbs	−0.219	0.068	.001	−0.305	0.068	<.001	September	16.25
US_Doppler in general	0.354	0.137	.009	0.125	0.135	.353	February	4.08
US_Doppler_upper_limbs	−0.163	0.163	.317	0.358	0.165	.030	May	2.98
US_Neck	0.107	0.079	.168	−0.379	0.078	<.001	October	13.1
US_Breast	0.284	0.061	<.001	−0.745	0.065	<.001	October	50.2
Ultrasound_3D	−0.926	0.108	<.001	0.300	0.098	.003	June	25.5
US_Pregnancy_fetal_anomaly	−0.485	0.350	.165	−0.316	0.344	.358	August	1.08
US_Anterior_Abdominal_Wall	0.227	0.256	.375	0.056	0.254	.825	February	0.067
US_Prostate	0.188	0.050	<.001	0.199	0.050	<.001	November	20.6
US_Soft_tissues	−0.182	0.238	.444	−1.13	0.274	<.001	October	5.40
US_Doppler_carotid	−0.374	0.235	.112	−0.501	0.239	.035	September	2.57
US_Doppler_testes	−0.023	0.165	.885	−0.197	0.166	.237	October	1.32
US_Legs	0.322	0.385	.402	−0.771	0.408	.059	November	1.38
US_Urinary_tract	0.041	0.423	.921	−1.18	0.495	.016	October	1.78
US_Hips	0.632	30.73	.090	0.472	0.365	.196	February	1.47
US_Pelvis_transvaginal	−0.404	0.326	.214	0.061	0.319	.848	July	0.81
US_Gallbladder	−0.682	0.477	.152	−1.18	0.527	.025	September	1.91
US_Doppler_renal	−1.57	0.610	.009	−0.686	0.500	.169	August	2.42
US_Kidney	−0.857	0.430	.045	0.135	0.395	.732	July	1.35
US_Pelvis	−0.079	0.068	.244	−0.164	0.068	.016	September	7.17
US_Adrenals	0.048	0.365	.893	0.048	0.365	.893	February	0.09
US_Axilla	0.119	0.296	.686	−0.207	0.297	.486	November	0.51
US_Abdomen_and_pelvis	0.116	0.020	<.001	−0.208	0.020	<.001	November	106.1
US_Abdomen_and_renal_tract	0.141	0.031	<.001	−0.293	0.031	<.001	September	66.1
US_Testes	−0.063	0.066	.336	−0.057	0.066	.383	August	3.39
US_Abdomen	0.162	0.019	<.001	0.019	0.019	.033	January	75.3
US_Thyroid	−0.092	0.119	.044	−0.098	0.119	.041	September	1.67
US_Groin_and_inguinal_region	0.161	0.168	.033	−0.378	0.171	.002	November	2.93

Generalized linear regression coefficients (b), robust standard error (SE), and probability of significance (*P*) for cosine and sine transformations of the scan month. The models were adjusted for age and gender. The seasonality of US requests was inferred from the significance (*P* < .05) of the cosine and/or sine generalized linear regression coefficients.

### 3.2. Tests of seasonality of US request (or order) priority

**Figure 5.**
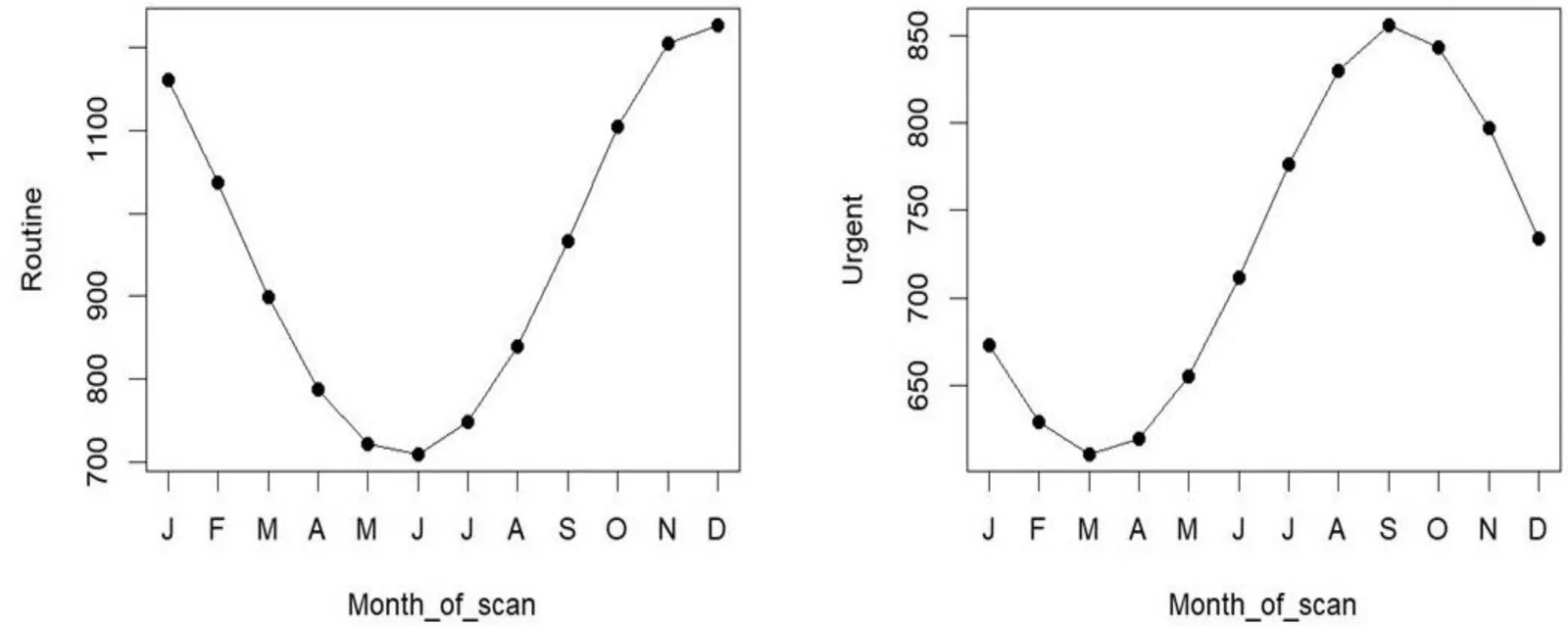
The seasonal pattern of US order-priority requests over the past 3 years. The data has been fitted with a cosinor model to highlight the sinusoidal pattern in the order-priorities throughout the seasons.

A cosinor GLM analysis was used to examine the presence of a sinusoidal pattern in the order of priority of US scans over the past 3 years (Table 3). We aimed to determine whether the inclusion of sine and/or cosine transformations of the month of scan improved the model fit compared to models that did not include such transformations. The results demonstrated significant cosinor terms for the order priority of US scans over the past 3 years (Fig. 5). Specifically, the acrophase peak for routine US cases was observed in December, whereas that for urgent cases occurred in September. Among the different priority orders, routine US cases exhibited the highest amplitude (Table 3).

**Table 3:** Cosinor parameters and model comparison for US order priority.

Outcome	Multivariate generalized regression estimates						Acrophase	Amplitude
US-order priority	Cosine coefficient	(SE)	*P*	Sine coefficient	(SE)	*P*	(Month)	
Routine cases	0.218	0.013	<.001	−0.168	0.013	<.001	December	296.5
Urgent cases	−0.071	0.015	<.001	−0.154	0.015	<.001	September	133.7

Generalized linear regression coefficients (b), robust standard error (SE), and probability of significance (*P*) for cosine and sine transformations of the scan month. The models were adjusted for age and gender. The seasonality of US requests was inferred from the significance (*P* < .05) of the cosine and/or sine generalized linear regression coefficients.

### 3.3. Associations of photoperiod with US requests and US order-priority

Multinomial regression analysis was conducted to examine the relationships between all US scan requests (adjusted for age and sex) and a continuous variable called photoperiod. The likelihood ratio test indicated a significant overall linear correlation between the variables (*P* < .001). A post hoc test further explored the specific correlations between the photoperiod and different US scan subtypes. The post hoc results revealed a positive correlation between photoperiod and the US 3D scan subtype, with a coefficient (B) of.999 and a standard error of 0.397 (B(233) = 1.01 ± 0.397), which was statistically significant (*P* < .001). A Bonferroni correction was applied to adjust the significance threshold to *P* < .001 (0.05, divided by 30 comparisons). Even after the correction, the correlation remained significant (*P* < .001). Additionally, binomial logistic regression analysis examined the relationship between US order priority and photoperiod, revealing a significant positive linear correlation. The coefficient (B) for this correlation was 0.294, with a standard error of 0.021 (B(20,094) = 0.294 ± 0.021), which was also statistically significant (*P* < .001).

## 4. Discussion

To our knowledge, the current study is the first to demonstrate the seasonality of US requests and its association with the photoperiod in the Jazan region. Our study aimed to investigate the seasonality of US requests and their association with the photoperiod. Analysis of a 3-year dataset revealed significant seasonal variations in scan volume and priority, with peak demand at different times of the year. The positive correlation between photoperiod and certain scan subtypes suggests that daylight duration may influence healthcare-seeking behavior.

Our study highlights the interplay among seasonality, disease patterns, and diagnostic practices in Jazan, offering insights into healthcare utilization. By examining US request seasonality and photoperiod correlations, we aimed to uncover trends in imaging use and factors driving disease dynamics. We will further explore the implications of our findings by comparing them with previous research and examining the causes of the observed patterns in disease and US scan types.

Seasonal variations in disease occurrence have been widely documented across medical disciplines, with studies demonstrating their impact on cardiovascular diseases, infectious diseases, and mood disorders.^[1–3]^ Our study corroborates these findings by revealing distinct seasonal patterns in US requests, aligned with previous research on healthcare utilization patterns.^[4]^ This consistency underscores the multifaceted influence of environmental, cultural, and biological factors on disease dynamics and diagnostic practice.

A notable finding of our study was the temporal clustering of US requests during specific months, particularly August and November, coinciding with peaks in various US scan subtypes. These temporal clusters may reflect seasonal changes in disease prevalence, clinical presentation, and healthcare-seeking behaviors. Previous research has linked seasonal variations in infectious disease transmission to an increased demand for diagnostic imaging services during certain months, highlighting the potential role of environmental factors in driving healthcare utilization patterns.^[2]^ Moreover, cultural and societal factors, such as awareness campaigns and healthcare policies, may contribute to observed temporal trends in US requests, as evidenced by studies on cancer detection rates in response to public health initiatives.^[15]^

Furthermore, our analysis revealed variations in the amplitude of seasonal fluctuations across different US scan subtypes, with the abdominal and pelvic scans exhibiting the highest amplitudes. These variations may stem from differences in the disease prevalence, clinical indications, or population demographics. For example, seasonal fluctuations in the severity of acute abdominal conditions have been documented, suggesting a potential link between environmental factors and abdominal pathology.^[16]^ Similarly, the observed seasonality of breast US requests may be influenced by hormonal fluctuations or screening campaigns targeting breast cancer awareness.^[15]^ Also, this is most probably due to the recognition of the third Friday in each October as “national mammography^[15]^ Interestingly, there were exceptions to this pattern, such as fetal anomaly US peaking in August and Doppler testicular requests peaking in October, indicating unique seasonal trends. Prior studies have investigated the relationship between diseases and seasonal patterns, notably the heightened severity of acute abdominal conditions during the summer.^[16]^ This study specifically examined variations in thyroid-stimulating hormone concentrations, considering all temperature-dependent factors, revealing significant variability. Understanding these fluctuations is imperative for precise diagnostic evaluation. Notably, thyroid-stimulating hormone levels peak in winter and dip in summer, exhibiting a negative correlation with temperature.^[16]^

The association between photoperiod and US scan requests adds another dimension to our findings, highlighting the role of daylight exposure in shaping healthcare utilization patterns. Photoperiods serve as a proxy for environmental cues that influence biological rhythms, mood, and behavior.^[2]^ Furthermore, the study found a significant relationship between the daylight period and the subtype of 3D US scans, even after applying the Bonferroni correction. Additionally, the prioritization of US scan procedures showed a seasonal pattern, with routine cases peaking in December and emergency cases in September, highlighting the need for effective scheduling and resource allocation. Moreover, multivariate logistic analysis showed interplay to estimate the relationship between request prioritization and daylight length, revealing a positive effect between immediate examination requests and daylight length. These insights provide healthcare managers and physicians with valuable information to improve processes and patient care management and potentially reduce wait times by predicting and addressing increasing demand periods for US examination services.

While previous research has explored seasonal variations in healthcare utilization for various medical services, including emergency department visits and hospital admissions^[14]^ our study contributes to the literature by specifically examining the seasonality of US requests in a unique geographical and cultural context. By incorporating photoperiod measurements, we offer a novel perspective on the influence of natural daylight duration on healthcare-seeking behavior, complementing existing studies on the impact of weather patterns and infectious disease incidence.

Comparing our findings with those of previous studies reveals both consistencies and disparities in the reasons and causes of diseases and variations in US scan types. While our study reaffirms the role of seasonal factors in shaping disease epidemiology and diagnostic practices, the unique demographic and environmental characteristics of the Jazan region introduce distinct nuances to healthcare utilization patterns. Understanding these contextual factors is crucial for designing effective interventions and healthcare delivery strategies tailored to the needs of local populations.

This study provides valuable clinical insight by identifying distinct seasonal patterns in US requests and correlating them with variations in photoperiod. The findings highlight that healthcare demand, particularly for abdominal, pelvic, and 3D ultrasound scans, is not consistent throughout the year, but instead peaks during specific months, such as August and November. Recognizing these temporal trends is crucial for optimizing healthcare service delivery. It allows radiology departments and hospital administrators to anticipate periods of increased imaging demand and adjust staffing levels, appointment scheduling, and resource allocation accordingly. Additionally, the observed seasonal variation in US order priority with routine scans peaking in December and urgent scans in September emphasizes the need for dynamic and flexible workflow management. Incorporating photoperiod aware planning into operational strategies may enhance diagnostic efficiency, reduce patient wait times, and improve overall care coordination. These insights are especially relevant for regions with similar environmental and demographic profiles and open the door to further research into environmental influences on healthcare utilization.

Our study provides insights into the seasonality of US requests and their correlation with photoperiod in the Jazan region, enhancing the understanding of healthcare utilization patterns. However, it is important to acknowledge these limitations and suggest that future research address these gaps. One limitation is the focus on the association between photoperiod and US scan requests, overlooking potential confounding factors, such as socioeconomic status, healthcare accessibility, and patient demographics, which could influence the observed trends. Additionally, relying on data from a single institution may limit the generalizability of our findings, as healthcare utilization patterns can vary across different facilities and populations. Consequently, our results may not be representative of broader trends beyond the Jazan region. Moreover, our analysis concentrated on the seasonality of US requests rather than the underlying diseases or clinical outcomes, providing only a partial understanding of the seasonal dynamics in disease prevalence and severity. Lastly, we did not explore other contextual factors such as environmental pollutants, dietary habits, or genetic predispositions, which may also contribute to seasonal variations in healthcare utilization. Investigating these factors could yield deeper insights into the trends observed in healthcare utilization and disease epidemiology.

## 5. Conclusion

Our study provides valuable insights into the seasonality of US requests and their association with the photoperiod in the Jazan region. By examining 3 years of data, we identified distinct temporal patterns in US utilization, with peaks observed during specific months, notably, August and November. These findings highlight the influence of environmental factors, particularly seasonal changes in the photoperiod, on diagnostic practices. In addition, our analysis revealed differences in the amplitude of seasonal fluctuations across various US scan subtypes, with the abdominal and pelvic scans exhibiting the highest amplitudes. This variation underscores the heterogeneity of the disease prevalence, clinical indications, and population demographics within the region.

## Author contributions

**Data curation:** Shaima A. Shanaq, Asmahan Y. Alhazmi, Abeer H. Maashi, Elaf A. Alsalem, Marwa A. Atiah.

**Formal analysis:** Wael A. Ageeli, Naif A. Majrashi.

**Investigation:** Wael A. Ageeli, Ali Y. Swedi, Turkey A. Refaee, Ali M. Tawhari, Shaima A. Shanaq, Asmahan Y. Alhazmi, Abeer H. Maashi, Elaf A. Alsalem, Marwa A. Atiah, Ali A. Hommadi, Mohammed O. Kuliby, Ali S. Alyami, Bandar A. Alwadani, Yahia Madkhali, Nouf Abohadi, Naif A. Majrashi.

**Methodology:** Wael A. Ageeli, Turkey A. Refaee, Naif A. Majrashi.

**Supervision:** Wael A. Ageeli, Turkey A. Refaee, Naif A. Majrashi.

**Writing – original draft:** Wael A. Ageeli, Naif A. Majrashi.

**Writing – review & editing:** Wael A. Ageeli, Ali Y. Swedi, Ali M. Tawhari, Shaima A. Shanaq, Asmahan Y. Alhazmi, Abeer H. Maashi, Elaf A. Alsalem, Marwa A. Atiah, Ali A. Hommadi, Mohammed O. Kuliby, Ali S. Alyami, Bandar A. Alwadani, Yahia Madkhali, Nouf Abohadi, Naif A. Majrashi.
